# Six-Month Outcomes of Post-ARDS Pulmonary Fibrosis in Patients With H1N1 Pneumonia

**DOI:** 10.3389/fmolb.2021.640763

**Published:** 2021-06-08

**Authors:** Jing Gao, Weili Chu, Jiali Duan, Junlu Li, Wentao Ma, Chunling Hu, Mengying Yao, Lihua Xing, Yuejie Yang

**Affiliations:** ^1^ Department of Respiratory Intensive Care Unit, The First Affiliated Hospital of Zhengzhou University, Zhengzhou, China; ^2^ Department of Respiratory Intensive Care Unit, The Sixth People’s Hospital of Zhengzhou, Zhengzhou, China

**Keywords:** ARDS, fibrosis, CT, H1N1 pneumonia, outcome

## Abstract

**Background:** Influenza virus is a common pathogen causing community-acquired pneumonia. After H1N1 infection, some patients present with rapid disease progression and various respiratory complications, especially immunocompromised patients and pregnant women. However, most patients have a favorable prognosis. Influenza viruses infect respiratory epithelial cells, leading to diffuse alveolar damage (DAD), which could induce secondary bacterial or fungal infections that could lead to serious complications, such as acute respiratory failure, severe pneumonia, pneumothorax, mediastinal emphysema, acute respiratory distress syndrome (ARDS) and post-ARDS fibrosis.

**Objective:** The short-term mortality rate of ARDS is decreasing, and understanding survivors’ posthospitalization outcomes is very important. Our aim was to evaluate the outcomes of 69 patients who survived H1N1 pneumonia with severe respiratory complications and abnormal CT findings and developed post-ARDS pulmonary fibrosis.

**Materials and methods:** The 280 inpatients included in this trial had been diagnosed with H1N1 infection that was confirmed by pharyngeal sputum or swab tests. The data were collected from January 2018 to January 2020 in the First Affiliated Hospital of Zhengzhou University and the Sixth People's Hospital of Zhengzhou. Of these patients, 232 had CT findings indicating pulmonary fibrosis after H1N1 infection, and 69 survived and consented to participate in this study. 6°months after diagnosis, the 69 surviving patients were interviewed and underwent physical examinations, CT scans, 6°min walk tests, and quality-of-life evaluations (SF-36). We analyzed the baseline variables and six-month outcomes of post-ARDS pulmonary fibrosis in patients with H1N1 pneumonia.

**Results:** Of the 69 surviving patients with post-ARDS pulmonary fibrosis, there were 24 females and 45 males, with a mean age of 53.7 ± 16.8°years; 18 patients (26%) had no underlying disease, and 14 (20%) patients had more than one underlying disease. The distance walked in 6°min increased from an average of 451.9°m at 3°months to 575.4°m at 6°months; the mean 36-Item Short Form Survey (SF-36) physical function score increased from an average of 75.3 at 3°months to 77.5 at 6°months; and the average CT score decreased from 31.3 at 3°months to 14.8 at 6°months. Treatment with systemic corticosteroids and the presence of an underlying disease were related to the CT score and the distance walked in 6°min.

**Conclusion:** Among the survivors with pulmonary fibrosis after H1N1 influenza, the 6°min walk test and CT scores continued to be affected after 6°months. The 6°min walk distance and imaging findings improved during the first 6°months. The health-related QoL (HRQoL) scores of H1N1 pneumonia survivors were lower than those of sex- and age-matched controls.

## Introduction

Influenza viruses, especially influenza A viruses (such as H1N1), cause a range of clinical syndromes, from self-limited disease to acute respiratory distress syndrome (ARDS) (Bautista et al., 2010). Complications due to the direct action of the virus or progression to a secondary infection with a poor clinical presentation could lead to ARDS, permanent fibrosis or death (Mukhopadhyay et al., 2010). Survivors of swine flu, especially those who developed ARDS, experienced long-term impairments in their physical, cognitive and mental health (Perez-Padilla et al., 2009; Takayama et al., 2011; Webb et al., 2011). To investigate the effects of ARDS, some researchers have used lung function tests, mental function scale scores, cognitive assessments, and quality-of-life measures (Chan et al., 2017; Latronico et al., 2017; Kacmarek and Berra, 2018; Schmidt et al., 2018), and most have indicated that there is persistent morbidity after discharge from the intensive care unit (ICU). Herridge MS and colleagues found that survivors of ARDS have persistent functional disability one year after discharge from the ICU. Most patients have extrapulmonary disease, with muscular atrophy and weakness being most prominent (Herridge et al., 2003).

The short-term mortality rate due to ARDS is decreasing, and understanding survivors’ posthospitalization outcomes is increasingly important. Therefore, the aim of this study was to characterize the long-term pulmonary and extrapulmonary function in a retrospectively identified cohort of patients who survived ARDS induced by H1N1 pneumonia.

## Methods

### Patients

This study was conducted at the ICUs of the First Affiliated Hospital of Zhengzhou University and the Sixth People’s Hospital of Zhengzhou from January 2018 to January 2020. We collected patients with a diagnosis of H1N1 infection confirmed by pharyngeal swab or sputum tests. Patients between the ages of 18 and 70 years who met the diagnostic criteria for ARDS were enrolled (ARDS Definition Task Force et al., 2012). The patients had factors predisposing them to ARDS, an oxygenation index (PaO_2_:FiO_2_ ratio) less than or equal to 200 or the use of mechanical ventilation with a positive end-expiratory pressure of at least 5 cmH_2_O, and chest radiographs with 3 or 4 quadrants containing opacities (ARDS Definition Task Force et al., 2012). The exclusion criteria included immunosuppression, malignant tumor (expected survival time less than 6°months), neuromuscular disease, liver failure, and severe neurologic disease. We obtained informed consent from all patients or their legal representatives. This study was approved by the institutional ethics committee of the two hospitals (ethics approval number is 2020-KY-446).

The Acute Physiology, Age and Chronic Health Evaluation (APACHE II) was used to assess the severity of illness within the first 24°hours after the patient was admitted to the ICU. We used the 36-Item Short Form Survey (SF-36) to measure health-related quality of life. The SF-36 includes eight scales: physical functioning, social functioning, role physical, role emotional, mental health, body pain, vitality, and general health (Lins and Carvalho, 2016).

### Data Collection

The following data were prospectively collected at the time of admission to the ICU: age, sex, chronic diseases, need for noninvasive or invasive ventilation, APACHE II score, need for corticosteroids, need for renal replacement therapy, duration of extracorporeal membrane oxygenation (ECMO) support, duration of renal replacement therapy, duration of ICU stay, and duration of hospitalization.

### Procedures Six Months After ICU Discharge

Patients underwent 3 and 6°month follow-up visits after discharge from the ICU. At each visit, all patients were interviewed about their symptoms and activities. Lung morphology was assessed with a chest high-resolution CT (HRCT) scan, and lung function was assessed with the 6°min walk test. Health-related QoL (HRQoL) was evaluated with the SF-36.

### HRCT Score

The HRCT findings were graded based on the classification system proposed by Ichikado et al. (Ichikado et al., 2002; Ichikado et al., 2006) as follows: areas with normal attenuation, ground-glass opacities (GGOs), consolidation, GGOs with traction bronchiectasis or bronchiolectasis, consolidation with traction bronchiectasis or bronchiolectasis, and honeycombing. Each lung was divided into three parts (upper, middle and lower), and each part was evaluated separately. The extent of each abnormality was determined by visually estimating the percentage of the affected lung in each part. The abnormality score for each part was calculated by multiplying the area percentage by the point value. The scores for the six parts were averaged to determine the total score for each abnormality in each patient. The overall CT score for each patient was obtained by adding the six averaged scores.

### Statistical Analyses

The primary outcomes of the study were the distance walked in 6°min and the CT score at 3 and 6°months after discharge from the ICU. These measurements provide standardized, objective, integrated assessments of the cardiopulmonary system that are relevant to daily activities. The nonnormally distributed continuous variables are shown as medians with interquartile ranges (IQRs). We performed univariate analysis and multivariate analysis to identify the potential determinants of long-term function, as reflected in the distance walked in 6°min and CT score. Tests were two-sided, and a P value less than 0.05 was considered statistically significant. Variables significant in the univariate analyses

(*P* < 0.1) were considered for inclusion in the multivariable linear regression analysis. The multivariate analysis was performed using backward stepwise selection for each follow-up period (3 and 6°months). All analyses were performed with SPSS 21.0 (SPSS, Chicago, IL).

## Results

### Characteristics of the Patients

**FIGURE 1:**
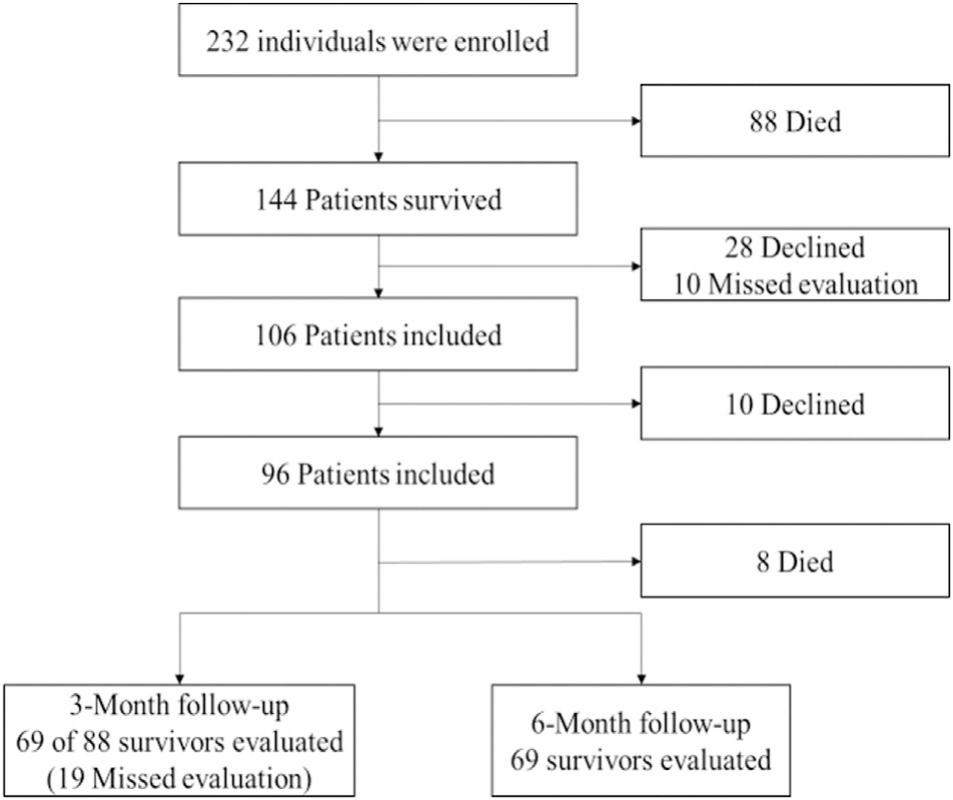
Enrollment of patients and follow-up for the first 6°months after discharge.

Over the 2°years, we recruited 280 patients, and 232 patients were included in the study. Of those 232 patients, 32 died, 16 refused to participate, and 144 survived to discharge. Of the 106 patients who remained alive at the time of follow-up, consent was obtained from 69, and these patients were included in the study (Figure 1).

All deceased patients died due to underlying diseases during the first 3°months after discharge from the ICU. Four patients died of multisystem organ failure, two patients died of cardiac arrest, one patient died of hepatic failure, and one patient died of hemoptysis.

The median age of the patients with ARDS who survived to discharge from the ICU was 55°years, and 65% were male (45/69). The APACHE II score reflects the severity of illness in these patients. This group of patients spent a median of 7°days in the ICU and 15°days in the hospital. Two patients required ECMO support, and three patients required renal replacement therapy in the ICU.

### Distance Walked in Six Minutes, Quality of Life, and the Relationships Between Patient Characteristics and Distance Walked in Six Minutes

The distance walked in 6°min improved over the 6°months after discharge from the ICU. The patients attributed exercise limitations to muscle wasting and weakness, weight loss, pain, and dyspnea. The scores for most domains on the SF-36 improved from 3 to 6°months after discharge from the ICU (Table 1). Compared to at 3°months, patients’ social functioning, mental health and pain were improved at 6°months. The difference was statistically significant (*P* < 0.05). The distance walked in 6°min improved dramatically during the 6°months after discharge, and the difference was statistically significant (*P* < 0.05).

**TABLE 1:** Ability to exercise and health-related quality of life among patients during the first 6°months after discharge.

Variables Median (IQR)	3°Months (*n* = 69)	6°Months (*n* = 69)	P*
Distance walked in 6°mins	461.0 (314.5–579.5)	587.0 (501.5–679.0)	<0.001
CT score	21.0 (8.0–39.5)	10.0(2.5–20.0)	<0.001
SF-36 score			
Physical functioning	80.0 (65.0–90.0)	85.0 (62.5–90.0)	0.395
Role physical	75.0 (50.0–100.0)	75.0 (12.5–100.0)	0.144
Body pain	62.0 (41.0–79.0)	90.0 (74.0–90.0)	<0.001
General health	47.0 (45.0–53.0)	52.0 (45.0–57.0)	0.111
Vitality	70.0 (60.0–77.5)	75.0 (65.0–85.0)	0.419
Social functioning	66.7 (55.6–77.8)	77.8 (66.7–88.9)	0.007
Role emotional	66.7(33.3–66.7)	66.7 (33.3–100.0)	0.813
Mental health	72.0 (56.0–82.0)	80.0 (68.0–88.0)	0.010

*Wilcoxon signed-rank test was used to compare the difference between the two groups.

IQR, Interquartile range; SF-36, 36-item Short Form Survey.

Then, we analyze the predictors of the distance walked in 6°min during the first 3°and 6°months after discharge. The results of univariate analyses are presented in Table 2. Compared with patients without underlying diseases, patients with underlying diseases had a significantly shorter distance walked in 6°min at both 3 (*P* < 0.001) and 6°months (*P* = 0.045). Treatment with systemic corticosteroids in the ICU, the presence of underlying diseases, the total days on ventilation, and the APACHE II score were the most important determinants of the distance walked in 6°min during the first 6°months of follow-up.

**TABLE 2:** Predictors of distance walked in 6°min during the first 6°months after discharge.

Variables	3°Months		6°Months	
	Univariate Analysis	Multivariate Analysis (*R* ^2^ = 0.486)	Univariate Analysis	Multivariate Analysis (*R* ^2^ = 0.314)
Age				
β coefficient	−5.451 ± 1.045	−5.415 ± 0.955	−4.376 ± 0.887	−4.191 ± 0.906
*P* value	＜0.001	＜0.001	＜0.001	＜0.001
Male sex				
β coefficient	70.125 ± 42.788	95.952 ± 33.043	4.644 ± 36.464	
*P* value	0.106	0.005	0.899	
BMI^Ψ^				
β coefficient	−3.781 ± 9.061		−5.265 ± 7.555	
*P* value	0.678		0.488	
Underlying disease				
β coefficient	−163.5 ± 39.869	−118.4 ± 34.274	−74.00 ± 36.158	−36.116 ± 33.202
*P* value	＜0.001	0.001	0.045	0.281
Systemic corticosteroid treatment				
β coefficient	56.000 ± 43.554		15.348 ± 36.798	
*P* value	0.203		0.678	
Length of ICU stay				
β coefficient	−0.802 ± 2.399		1.544 ± 1.998	
*P* value	0.739		0.442	
Duration of hospitalization				
β coefficient	0.831 ± 1.377		1.741 ± 1.134	1.438 ± 1.180
*P* value	0.548		0.129	0.227
Total days of ventilator use				
β coefficient	5.302 ± 3.672	0.261 ± 2.831	5.010 ± 3.055	0.238 ± 3.200
*P* value	0.153	0.927	0.106	0.941
APACHE Ⅱ score#				
β coefficient	−4.123 ± 3.879		−2.794 ± 3.252	
*P* value	0.292		0.393	

*Plus-minus values are standard errors.

^#^Scores for the Acute Physiology, Age, and Chronic Health Evaluation (APACHE II) can range from 0 to 71; higher scores indicate more severe illness.

^Ψ^BMI indicates body mass index (the weight in kilograms divided by the square of the height in meters), which was calculated for each period.

The results of multivariate analyses are also presented in Table 2. Patients with underlying diseases had a shorter distance walked in 6°min than those without underlying diseases at 3°months (*P* = 0.001) but not at 6°months (*P* = 0.281). The absence of an underlying disease during the stay in the ICU had the strongest association with a longer distance walked in 6°min at 3°months (R^2^ = 0.486). There was no factor during the ICU stay that was associated with a longer distance walked in 6°min at 6°months (R^2^ = 0.314). A longer duration of hospitalization was associated with a shorter distance walked in 6°min at 6°months but not at 3°months.

### Chest Radiography and the Relationships Between Patient Characteristics and the CT Score

**FIGURE 2:**
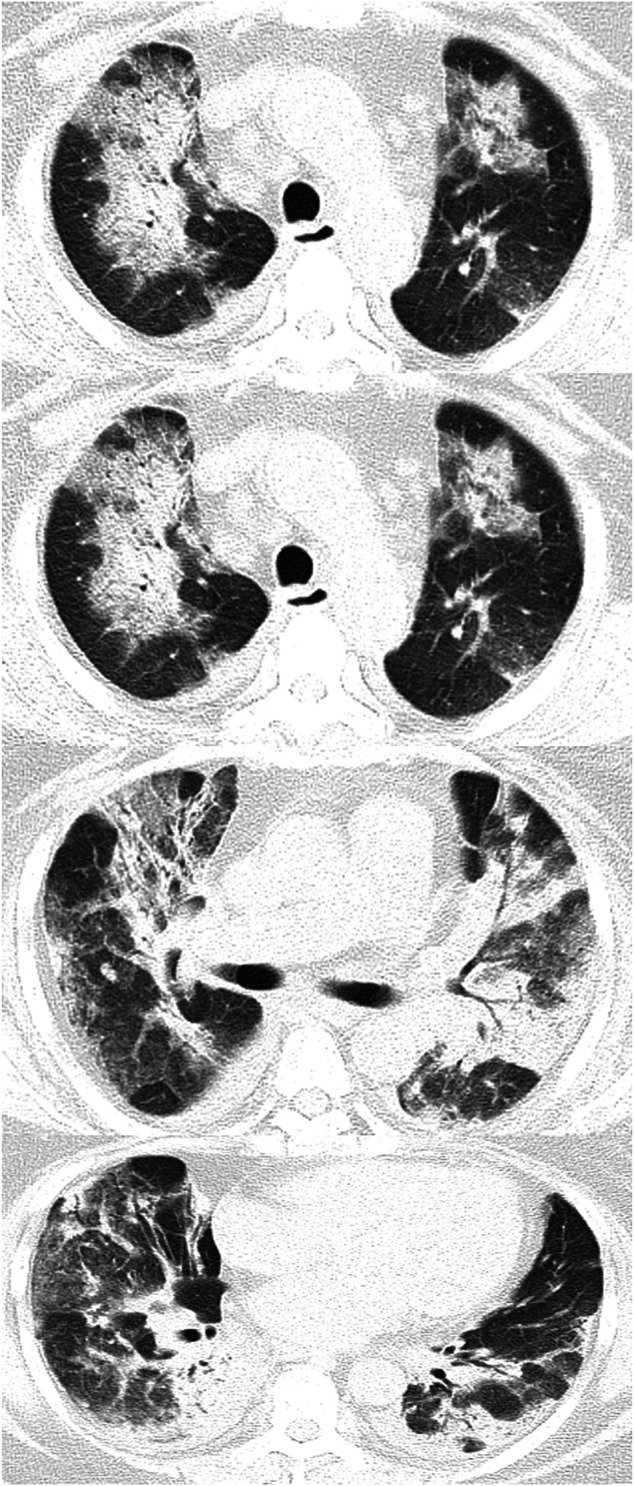
These scans are of a 64°year-old woman with an H1N1 infection confirmed by a sputum test. Chest CT scan demonstrates patchy shadows and ground-glass opacities in both lungs, with traction bronchiectasis and pleural thickening.

Chest radiography showed the presence of GGOs, traction bronchiectasis and consolidation (Figure 2). Radiologic changes included the lower line of the pleura and isolated areas of pleural thickening at 3°months. At 6°months after discharge from the hospital, all but 4 patients showed improvement on chest CT. The CT score improved dramatically during the 6°months, and the difference was statistically significant (*P* < 0.05) (Table 1).

Then, we analyzed the predictors of the CT score in the first 3 and 6°months after discharge. The results of univariate analyses are presented in Table 3. Consistent with the result regarding the distance walked in 6°min, the presence of underlying diseases was the most important determinant of the CT scores at 3 (*P* = 0.002) and 6°months (*P* < 0.001).

**TABLE 3:** Predictors of the CT score during the first 6°months after discharge.

Variables	3°Months		6°Months	
	Univariate Analysis	Multivariate Analysis (*R* ^2^ = 0.364)	Univariate Analysis	Multivariate Analysis (*R* ^2^ = 0.282)
Age				
β coefficient	0.351 ± 0.257	0.310 ± 0.228	0.311 ± 0.116	0.230 ± 0.109
*P* value	0.178	0.180	0.009	0.038
Male sex				
β coefficient	−13.289 ± 9.237	−13.429 ± 8.051	0.758 ± 4.298	
*P* value	0.146	0.100	0.860	
BMI				
β coefficient	0.006 ± 1.909		−0.275 ± 0.893	
*P* value	0.998		0.759	
Underlying disease				
β coefficient	28.183 ± 8.729	25.108 ± 8.155	17.025 ± 3.871	15.209 ± 3.960
*P* value	0.002	0.003	＜0.001	＜0.001
Systemic corticosteroid treatment				
β coefficient	5.587 ± 9.252		2.283 ± 4.335	
*P* value	0.548		0.600	
Length of ICU stay				
β coefficient	0.971 ± 0.491	2.435 ± 0.567	0.104 ± 0.236	
*P* value	0.052	＜0.001	0.662	
Duration of hospitalization				
β coefficient	0.266 ± 0.289		−0.047 ± 0.136	
*P* value	0.359		0.731	
Total days of ventilator use				
β coefficient	−0.767 ± 0.779	−2.686 ± 0.931	−0.491 ± 0.362	−0.168 ± 0.363
*P* value	0.328	0.005	0.180	0.645
APACHE II score				
β coefficient	0.376 ± 0.822	0.095 ± 0.772	0.074 ± 0.385	0.196 ± 0.368
*P* value	0.648	0.902	0.849	0.596

*Plus-minus values are standard errors.

^#^Scores for the Acute Physiology, Age, and Chronic Health Evaluation (APACHE II) can range from 0 to 71; higher scores indicate more severe illness.

^Ψ^BMI indicates body mass index (the weight in kilograms divided by the square of the height in meters), which was calculated for each period.

The results of multivariate analysis are also presented in Table 3. Patients with underlying diseases had higher CT scores at 3 (*P* = 0.003) and 6°months (*P* < 0.001) than those without underlying diseases. A longer ICU stay and duration of ventilator use were associated with a higher CT score at 3°months but not at 6°months.

## Discussion

Influenza viruses, of which type A influenza viruses are the most common, can cause pandemics. Type A influenza viruses are characterized by a high mutation rate, leading to greater virulence. The H1N1 virus, which first appeared in Mexico in April 2009, has spread rapidly around the world, raising new concerns about a disease that has had high death rates in the past (Hui et al., 2010). Influenza A (H1N1) infection can lead to severe respiratory illness. ARDS is a major cause of influenza-related deaths, with a high incidence in severe cases (Jaber et al., 2010; Ramsey and Kumar, 2011; Webb et al., 2011). We found that patients who survived ARDS had persistent functional limitations 6°months after discharge from the hospital, largely as a result of muscle wasting, weakness and social functioning and mental health deficits.

Many studies of patients with ARDS have focused on the effects on the lungs and have shown that lung function returns to normal or near normal within 6°months–1°year. Those who do not recover within that timeframe are generally those with lung damage who had long stays in the hospital and ICU. These patients often experience chronic cognitive and mental illnesses (Mikkelsen et al., 2012), reductions in their physical function and QoL, negative effects on employment (Griffiths et al., 2013), and increased costs and use of health care services (Herridge et al., 2011). At the same time, the HRQoL of survivors is significantly lower than that of the general population, and survivors may have social functioning and mental health deficiencies (Weinert et al., 1997). In these survivors, the affected domains of HRQoL are predominantly role physical, bodily pain, social functioning and role emotional. In our study, the scores on most domains of the SF-36 improved from 3to 6°months after discharge from the ICU (Table 1). The scores for domains reflecting pain and reported health transitions improved dramatically during the first 6°months of follow-up, paralleling the incremental improvement in the distance walked in 6°min. The distance walked in 6°min improved over the 6°months after discharge from the ICU but remained lower than the predicted value. The 6°min walk test and QoL assessments were consistent with data published previously. A meta-analysis showed that the HRQoL of ARDS survivors recovered during the first 6°months after discharge (Dowdy et al., 2006). Margaret S and colleagues noted that the scores on all domains of the SF-36 improved from 3 to 6°months after discharge from the hospital. The scores for the physical role and physical functioning domains improved dramatically, paralleling the incremental improvement in the distance walked in six°min (ARDS Definition Task Force et al., 2012). Chan and colleagues found that some measures of activity, especially the 6°min walk test, were associated with HRQoL, but this was not consistent (Latronico et al., 2017). Impaired muscle function may explain the compromised functional ability and QoL in our cohort and those studied by other investigators.

In patients with H1N1 pneumonia, post-ARDS pulmonary fibrosis is not a rare complication. Therefore, a follow-up CT is very important. CT showed that pulmonary fibrosis improved over time, especially in the first 6°months after discharge (Chen et al., 2017). However, Bai L and his team found that with regard to the long-term prognosis of severe ARDS associated with the 2009 H1N1 pandemic, the imaging findings in patients one year after discharge from the ICU remained abnormal and were characterized by interlobular septal thickening, fibrous cords, and traction bronchiectasis (Bai et al., 2011). In another study, 85.7% of the patients had GGOs at 3°months of follow-up (Luyt et al., 2012). In a univariate analysis (Table 2), we found that treatment with systemic corticosteroids during admission to the ICU and the presence of underlying diseases were the most important determinants of CT score during the first 6°months of follow-up. There has been great interest in the role of corticosteroids in attenuating pulmonary and systemic damage in patients with ARDS because of their potent anti-inflammatory and antifibrotic properties (Rhen and Cidlowski, 2005). Different regimens of corticosteroids have been tested in ARDS, but the results have been inconclusive (Meduri et al., 1998; Annane et al., 2006; Steinberg et al., 2006; Meduri et al., 2007). Guidelines recently issued by the Society of Critical Care Medicine and the European Society of Intensive Care Medicine provide conditional recommendations with a moderate quality of evidence for the administration of glucocorticoids based on a meta-analysis of nine randomized controlled trials in ARDS patients (Annane et al., 2017). Despite the lack of conclusive results, it remains clinically and biologically plausible that corticosteroids could benefit patients with ARDS in the early phase of the disease, a situation that has not been evaluated in most randomized controlled trials.

To our knowledge, this is the first retrospective study of the physical and mental health of patients with H1N1 pneumonia during convalescence. This study had several limitations. First, follow-up visits were offered to all patients discharged from the hospital, but some refused to attend, and some did not complete follow-up. Therefore, we do not have data on lung function in those patients. Although many indices did not change significantly after 6°months, the study population may not be representative of the entire population of H1N1 survivors. Second, this was a retrospective study on the impact of H1N1 infection on the physical and psychological health of survivors, and no information on the baseline lung function and QoL of these patients was available. Therefore, we cannot compare the index of lung function before and after H1N1 infection. In particular, this group of patients had pre-existing conditions, which may also have affected their HRQoL. Finally, it is not clear whether further improvements in physical and mental health could be detected over a longer duration of follow-up. Therefore, the scope of research needs to be further expanded.

In summary, survivors of H1N1 pneumonia still have long-term pulmonary and psychological damage 6°months after discharge from the hospital. However, the 6°min walk test and the imaging findings improved in the first 6°months, especially in ARDS patients. The HRQoL scores of H1N1 pneumonia survivors were impaired and lower than those of sex and age-matched controls.

## Data Availability

The raw data supporting the conclusions of this article will be made available by the authors, without undue reservation.
